# Perimembranous Ventricular Septal Defect Concurrent with an Aorto-Right Ventricular Fistula Caused by a Ruptured Sinus of Valsalva in Three Dogs

**DOI:** 10.3390/ani15070944

**Published:** 2025-03-25

**Authors:** Juyeong Kim, Won-Jong Lee, Youngwon Lee, Hojung Choi, Dae-Hyun Kim

**Affiliations:** 1 Research Institute of Veterinary Medicine, College of Veterinary Medicine, Chungnam National University, Daejeon 34134, Republic of Korea; jyhsi@naver.com (J.K.); lywon@cnu.ac.kr (Y.L.); 2 Department of Veterinary Surgery, College of Veterinary Medicine, Chungnam National University, Daejeon 34134, Republic of Korea; wjl03ssaaa@gmail.com

**Keywords:** congenital cardiac disease, continuous murmur, aneurism, canine

## Abstract

An aorto-right ventricular fistula, a rare condition in humans, is characterized by communication between the ascending aorta and the right ventricle through a defect in the aortic wall. This report describes three cases of dogs with continuous murmurs: a 6-month-old Coton de Tulear, a 5-year-old Maltese, and a 6-month-old Jindo. Notably, all of the dogs presented with no severe clinical signs. Echocardiography revealed a turbulent jet through restrictive perimembranous ventricular septal defects (VSD) during systole and aorto-right ventricular fistulas secondary to ruptured sinuses of Valsalva aneurysm during diastole. In one case, a surgical closure of the VSD simultaneously resolved the aorto-right ventricular fistula. Follow-up echocardiography in the other two cases revealed mild left heart volume overload and a slight increase in the pulmonary-to-systemic blood flow ratio. However, the dogs remained asymptomatic. In conclusion, aorto-right ventricular fistulas with VSDs should be considered in the differential diagnosis of continuous murmurs in dogs.

## Simple Summary

An aorto-right ventricular fistula (ARVF) is a rare heart condition in dogs, characterized by an abnormal connection between the aorta and the right ventricle. This report describes three dogs diagnosed with an ARVF secondary to a ruptured sinus of Valsalva aneurysm, all of which also had ventricular septal defects (VSDs). Echocardiography revealed turbulent blood flow through the VSD during systole and through the ARVF during diastole. A surgical closure of the VSD successfully resolved the ARVF in one case, while the other two dogs remained asymptomatic with mild heart enlargements on follow-up. These findings highlight the importance of considering ARVF and VSD in dogs with continuous murmurs.

## 1. Introduction

An aorto-right ventricular fistula (ARVF) is a rare cardiac anomaly characterized by an abnormal connection between the ascending aorta and the right ventricle [1]. The abnormal communication allows blood to flow directly from the high-pressure aorta into the lower-pressure right ventricle, potentially leading to heart failure if left untreated [2]. A ventricular septal defect (VSD) is a congenital cardiac anomaly that can cause abnormal flow between the interventricular septum, usually leading to right ventricular overload [3]. VSDs are classified according to their location within the interventricular septum. The perimembranous VSD is an extended form of membranous VSD, typically located within the membranous portion of the ventricular septum [4]. This case report discusses the clinical presentation of ARVF with perimembranous VSD in a veterinary patient.

## 2. Materials and Methods

This study included three dogs diagnosed with ARVFs. The dogs underwent physical examination, blood tests, and thoracic radiography (XPLRER-900; GE Healthcare, Chicago, IL, USA), and echocardiography (Vivid E90, GE ultrasound systems, Boston, MA, USA) with phased array probes (GE 6S-D probe 2.4–8.0 MHz; GE Healthcare, Chicago, IL, USA) were performed. Other comorbidities, especially congenital cardiac diseases like patent ductus arteriosus, pulmonary stenosis, and atrial septal defect were excluded by thorough echocardiographic examination.

### 2.1. Case 1

A 6-month-old intact male Coton de Tulear dog weighing 6.5 kg was referred for VSD closure surgery (Table 1). The dog had a history of cough and exercise intolerance since 2-month-old and was administered spironolactone (1 mg/kg), enalapril (0.25 mg/kg), and ivabradine (0.25 mg/kg) twice daily due to sinus tachycardia. On physical examination, a grade 5 continuous murmur was heard at the right 5–6th intercostal space of the heart apex, with normal lung auscultation. Blood tests revealed no abnormalities (Table 2), while thoracic radiography revealed mild cardiomegaly (vertebral heart scase 11.0; reference range, 8.7–10.8).

**Figure 1:**
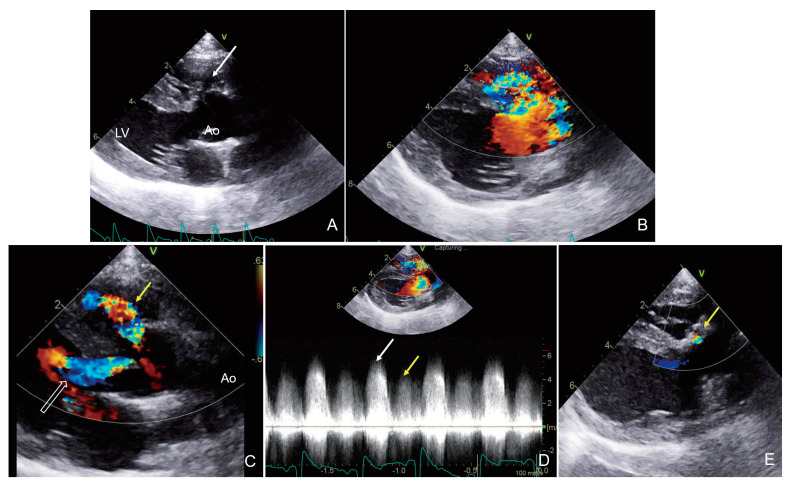
Echocardiogram images obtained from case 1. A small VSD occurred just below the aortic valve on the right parasternal left ventricular outflow view (white arrow) (**A**). Color Doppler examination showed systolic jet from the left ventricle to right ventricle (**B**), and aortic regurgitation (hollow arrow) and aorto-right ventricular fistula during diastole (yellow arrow) (**C**). Continuous wave Doppler examination revealed systolic left-to-right shunt through the VSD (white arrow) and diastolic shunt flow through the ruptured sinus of Valsalva aneurysm (yellow arrow) (**D**). After the VSD’s surgical correction, the flow in the fistula swirled only at the SOVA level (yellow arrow) and was not directed toward the RV (**E**). VSD: ventricular septal defect.

Two-dimensional (2D) echocardiography showed a 3.2 mm a perimembranous VSD beneath the aortic valve (Figure 1). A sinus of Valsalva aneurysm (SOVA) at the right aortic sinus was seen projecting into the right ventricle (RV) (Figure 1). Normalized left ventricular internal dimension in diastole (LVIDdN 1.70; reference range, 1.27–1.85) was within the reference range. The left atrium (LA) size was normal (left atrial-to-aortic ratio [LA/Ao], 1.4; reference range, <1.6). Color-flow Doppler imaging revealed a turbulent jet in the RV during systole and another jet during diastole from the left-to-right shunt across the VSD and SOVA, respectively. In addition, severe aortic regurgitation with a peak velocity of 4.1 m/s was observed as a turbulent jet flow extended into the left ventricle (LV) during diastole (Figure 1). Continuous wave Doppler examination showed 5.0 m/s of peak systolic flow velocity, suggesting a restrictive VSD, and a peak velocity of 4.3 m/s through the ruptured SOVA during diastole. A pulmonary-to-systemic blood flow ratio (Qp:Qs) of 2.0 was considered the upper limit of hemodynamic significance (Figure 1).

Based on these findings, the dog was diagnosed with a perimembranous VSD with an accompanying ARVF caused by a ruptured SOVA and severe aortic regurgitation. Based on this, the owner agreed to the surgical closure of the VSD using an autologous pericardial patch. Ivabradine and enalapril were gradually tapered during the week before surgery and stopped without any complications. During surgery, the patient was placed in the left lateral recumbent position, and a right thoracotomy was performed in the 5th intercostal space. A partial pericardiectomy was performed, and the excised pericardium was prepared for use as an autologous pericardial patch. The VSD was assessed through right atriotomy. A defect was detected on the right side, adjacent to the tricuspid valve. The VSD was closed with the two layers of glutaraldehyde-treated autologous pericardium by suturing with a continuous 5–0 polypropylene suture (Prolene; Ethicon, Johnson & Johnson Company, Raritan, NJ, USA). The right auricle was closed in a double simple continuous pattern using a 5–0 polypropylene suture (Prolene; Ethicon, Johnson & Johnson Company, Raritan, NJ, USA). The aortic valve was left untreated. Post-surgery, the systolic cardiac murmur disappeared, and only the diastolic murmur caused by aortic regurgitation and ARVF was heard. Echocardiography confirmed the closure of the perimembranous VSD 3 d post-surgery; this was confirmed by the disappearance of the systolic jet in the RV. In addition, the flow in the fistula swirled only at the SOVA level and was not directed toward the RV, as it had been corrected with the VSD closure. At 8 months post-surgery, echocardiography showed normal LVIDdN and LA/Ao ratios, and there was no evidence of LV enlargement or pulmonary hypertension. Aortic regurgitation persisted on Color-flow Doppler imaging. However, the cough and exercise intolerance had resolved, and sinus tachycardia was no longer present. Also, all of the medications were stopped immediately after surgery.

### 2.2. Case 2

A 5-year-old castrated male Maltese dog weighing 4.2 kg was referred for evaluation for a continuous murmur suspected to be a patent ductus arteriosus (PDA) (Table 1). The dog was asymptomatic, and no treatment had been administered. On physical examination, a grade 5 continuous murmur was heard at the right 4–5th intercostal space at the heart apex, with normal lung auscultation. Blood tests, including troponin and pro-brain natriuretic peptide levels, showed no abnormal findings (Table 2). Thoracic radiography showed mild cardiomegaly (ventricular hypertrophy score 11.1; reference range, 8.7–10.8).

**Figure 2:**
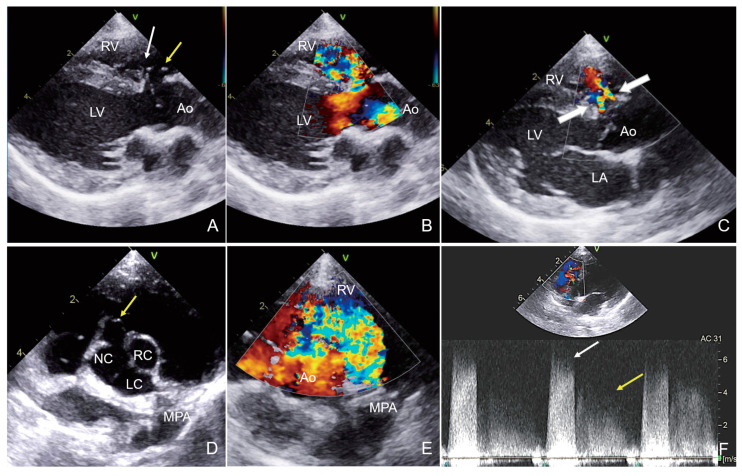
Echocardiogram images obtained from case 2. On a 2D echocardiogram, the VSD appeared very restrictive (white arrow in (**A**)) and a wind-sock appearance (yellow arrows in (**A**,**D**)) of the ruptured sinus of Valsalva aneurysm was visualized in the right parasternal LV outflow (**A**) and at the right parasternal short-axis aortic valve level (**D**). Color Doppler echocardiography showed systolic left-to-right shunt flow through a small ventricular septal defect (**B**,**E**). Two color flow streams from the aorta to the right ventricle (white arrow in (**C**)) were observed (**C**), and the continuous-wave Doppler spectrum of these streams was displayed during diastole (yellow arrow in (**F**)) after the systolic high-velocity (white arrow in (**F**)) VSD flow (**F**). VSD: ventricular septal defect. NC: non-coronary cusp; LC: left coronary cusp; RC: right coronary cusp.

Notably, 2D echocardiography revealed a 2.6 mm VSD immediately below the aortic valve in the right parasternal LV outflow view. The defect opened just below the tricuspid valve on the right side of the septum, indicating a perimembranous VSD (Figure 2), and a small aneurysmal dilation was suspected around the defect (Figure 2). The LVIDd was moderately increased (LVIDdN 1.96; reference range, 1.27–1.85), and the LA size was within the normal range (LA/Ao 1.30; reference range, <1.6). Color-flow Doppler imaging revealed a left-to-right shunt through the defect during systole, with a systolic peak flow velocity of 6.6 m/s, suggesting a restrictive VSD. In addition, two streams of turbulent jets into the RV from the sinus of Valsalva during diastole were detected (Figure 2). Continuous wave Doppler estimated a peak velocity of 5.1 m/s in the early diastole, which decreased gradually in the mid-to-late diastole. Due to the limitations of transthoracic echocardiography, the Doppler blood flow profile could not be fully recorded throughout the cardiac cycle. The pulmonary-to-systemic blood flow ratio (Qp:Qs) was 1.5, and there was no evidence of pulmonary hypertension.

Based on these findings, the dog was diagnosed with a perimembranous VSD concurrent with ARVF caused by SOVA with multiple fenestrations. Surgery was not performed because the dog was asymptomatic, and the Qp/Qs ratio was insignificant. The dog underwent follow-up recheck echocardiography after 8 months and was still asymptomatic. On echocardiography, the LA/Ao ratio (1.30) was still within the normal range, but the LVIDdN (2.06) and Qp:Qs (2.1) were increased and hemodynamically significant compared to the initial examination.

### 2.3. Case 3

A 6-month-old intact female Jindo dog weighing 7.1 kg was referred from a local animal hospital with a suspected VSD (Table 1). The patient had no clinical signs or history of prior treatment. On physical examination, a grade 6 continuous murmur was heard at the right 5–6th intercostal space at the heart apex with a normal lung auscultation. Blood tests revealed no abnormalities (Table 2) and thoracic radiography revealed mild cardiomegaly (ventricular hypertrophy score 11.1; reference range, 8.7–10.8).

**Figure 3:**
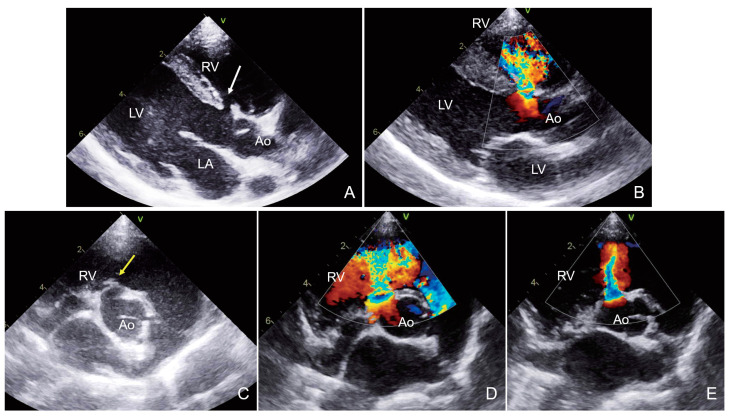
Echocardiogram images obtained from case 3. On the right parasternal left ventricular outflow view (**A**), a small VSD (white arrow) was clearly visualized. Wind-sock appearance of the ruptured sinus of Valsalva (yellow arrow) was shown on the right parasternal short axis aortic valve level (**C**). On the color Doppler examinations, systolic (**B**,**D**) and diastolic (**E**) turbulent flow was continuously observed.

Notably, 2D echocardiography showed a 4.3 mm VSD beneath the aortic valve. The shunt communicated with the RV just below the tricuspid valve, indicating a perimembranous VSD (Figure 3). In addition, the ruptured SOVA showed a “wind-sock” appearance in the right parasternal short axis aortopulmonary window (Figure 3). Due to the limitations of transthoracic 2D echocardiography, the aneurysm was not visualized in the right parasternal left ventricular outflow tract view. During systole, color-flow Doppler imaging revealed a left-to-right shunt through the ventricular septal defect. The peak systolic flow velocity (5.6 m/s) indicated a restrictive VSD. The LVIDdN (1.91; reference range, 1.27–1.85) was mildly increased. The LA/Ao ratio (1.60; reference range, <1.6) was at the upper margin of the normal range. The Qp:Qs ratio was 1.7, indicating low hemodynamic significance. In addition, color-flow Doppler imaging revealed a fistula at the sinus of Valsalva, with the turbulent flow directed toward the RV (Figure 3). The turbulent flow was observed throughout the diastole. During this examination, the peak velocity of the fistula was not recorded accurately.

Based on these findings, the dog was diagnosed with a perimembranous VSD accompanied by an ARVF due to a ruptured SOVA. Surgery was not performed at the owner’s discretion, as the patient was asymptomatic.

At the 8-month follow-up, the dog was asymptomatic. However, a physical examination revealed a grade 6 continuous murmur. Compared with the previous echocardiographic examination, mild left atrial volume overload (LA/Ao 1.87) and mildly increased pulmonary-to-systemic blood flow ratio (Qp:Qs 1.92) were identified in the dog. It was decided that the patient would undergo serial echocardiography every 6 months because it was currently asymptomatic. However, the follow-up has been lost.

## 3. Discussion

An ARVF is a communication between the ascending aorta and the RV through a defect in the aortic wall [1,5]. However, this condition is rarely reported in humans. It often occurs secondary to complications of trauma or infective endocarditis and may occur following surgical procedures, such as an aortic dissection or VSD repair [1,5,6,7]. An ARVF may be caused by congenital factors in patients with none of the underlying causes mentioned above [8,9,10]. In humans, some observational studies have shown that up to 76% of aorto-cardiac fistulas are due to a ruptured SOVA [11]. A SOVA is caused by weakness at the aortic media junction and the annulus fibrosus and most frequently originates from the right coronary cusp [12]. In addition, the estimated incidence of SOVA in the general population is approximately 0.09%, with ruptures occurring in 34% of those patients [13]. A SOVA can rupture into any cardiac chamber. However, the most common site is the RV [14]. A SOVA is often associated with other congenital cardiac defects, particularly VSDs [15]. In humans, less common cardiac lesions include aortic insufficiency and right ventricular outflow tract obstruction [16].

Echocardiography was the first diagnostic imaging modality used for screening congenital heart defects. The ruptured SOVA showed a “wind-sock” appearance, which is an elongated, tubular structure expanding into the receiving chambers on 2D echocardiographic images [13]. However, diagnosing a ruptured SOVA using only 2D images can be challenging. Sometimes, the wind-sock appearance of a ruptured SOVA cannot be visualized, and the presence of tricuspid valve tissue or a prolapsed aortic valve leaflet trapped within a VSD can show a similar “wind-sock” appearance [16]. VSDs are located in the interventricular septum underneath the aortic or pulmonic valve, whereas ruptured SOVAs originate at the sinus level above the aortic annulus. However, VSDs and SOVAs are located adjacent to each other and could be misdiagnosed due to probe manipulation and cardiac movement during examination [16].

Therefore, a ruptured SOVA should be differentiated from a VSD using color or continuous wave Doppler patterns. A VSD flow shunts from the LV to the RV with a high velocity during systole. However, similar to the PDA Doppler profile, the flow from a ruptured SOVA is a continuous turbulence with a high systolic velocity and a decelerating diastolic component from the aorta to the RV throughout the cardiac cycle. A SOVA is also mistaken for a membranous septal aneurysm.

However, in the right parasternal LV outflow view, usually used for diagnosing VSDs and aortic regurgitations, continuous flow from a ruptured SOVA cannot be visualized or is masked by VSD flows in color and continuous wave Doppler mode. In humans, transesophageal echocardiography can help image the flow from a ruptured SOVA, especially in the mid-esophageal aorto-vascular long-axis, short-axis, and RV inflow-outflow views [16,17]. Therefore, differentiating VSDs from ruptured SOVAs might be more challenging in dogs because of their small hearts and close anatomical locations.

In this study, the SOVA of the three dogs was considered as perimembranous septal aneurysms, which appeared as a thin membrane protruding into the RV from the margins of the VSD. It was either intact or perforated and did not show diastolic jet flow, even leading to spontaneous closure of the VSD [18,19]. However, continuous and color Doppler imaging showed abnormal diastolic flow into the RV in these cases, and an ARVF secondary to the ruptured SOVA was diagnosed.

When a SOVA ruptures, the left-to-right shunt becomes significant [13]. As the clinical signs worsen, patients may experience symptoms of acute heart failure, hemodynamic compromise, cardiac tamponade, and even sudden cardiac death [12]. However, all the dogs in this study were asymptomatic with mild cardiomegaly on the thoracic radiographs. Follow-up echocardiography revealed no significant changes in the fistulas, with no evidence of left-sided heart failure. It is thought that an acute event of rupture is not fatal, and a persistent shunt results in a chronic process. Case 2 and 3 did not undergo surgical correction, and the echocardiographic results after 8 months showed mild but progressive left ventricular volume overload. This eventually can result in increased pulmonary hypertension and left heart failure.

Continuous murmurs are commonly observed in humans and dogs with PDAs. Rarely, aortic regurgitation with a VSD and aorto-cardiac fistula can cause continuous murmurs [15]. During the echocardiographic examination, it is easy to overlook the presence of a ruptured SOVA if a VSD concurred with aortic regurgitation (which can explain the continuous heart murmur) and is imaged on the right parasternal LV outflow view.

The surgical treatment of an ARVF is usually recommended for humans. If diagnosed and treated promptly, significant morbidity resulting from congestive heart failure caused by a left-to-right shunt can be prevented [20]. Fistula closure is recommended, even in asymptomatic patients [10]. The incidence of surgical complications and the risks of heart failure, bacterial endocarditis, pulmonary vascular disease, aneurysm formation, and spontaneous rupture are low [10]. In a previous report, a supracristal VSD was closed using a pliable collagen bioscaffold patch, and the ARVF was repaired alongside [15]. In the first case in our report, surgical closure of the VSD using an autologous pericardial patch corrected the VSD and the ARVF due to the adjacent locations.

Our report has some limitations. First, computed tomography and cardiac catheterization angiography were not performed. Oftentimes, these examinations are performed in humans to confirm the diagnosis and delineate the coronary artery anatomy [21,22,23]. Second, surgical treatment was not confirmed in two of the dogs.

## 4. Conclusions

A VSD diagnosis is typically confirmed by observing the left-to-right jet flow in the right parasternal left ventricular outflow tract view. However, considering our cases, it was necessary to carefully examine the diastolic flow adjacent to the VSD using color-flow Doppler imaging. A VSD with a concurrent aortic regurgitation or ARVF secondary to a ruptured SOVA should be considered as a differential diagnosis, especially if the dogs presenting with continuous murmurs do not have typical findings of PDAs on radiography and echocardiography.

## Figures and Tables

**Table 1:** Echocardiographic measurements of cardiac function factors.

	Case 1		Case 2		Case 3
**Signalments**					
Breed	Conton de Tulear		Maltese		Jindo
Age	6 months		5 years		6 months
Sex	M		CM		F
Body Weight (kg)	6.5		4.2		7.1
**Date**	Day 1	After 8 ms	Day 1	After 8 ms	Day 1
**Radiography**					
VHS (v)	11	10.8	11.1	11.4	11.1
**Echocardiography**					
LA/Ao ratio	1.4	1.4	1.3	1.3	1.6
IVSd (mm)	7.1	7.6	7.3	6.7	8.8
LVIDd (mm)	25.3	30.9	30	32.4	34
LVPWd (mm)	5.5	6.9	5.4	7.2	5.5
IVSs (mm)	8.2	11.5	11.2	9.9	14.9
LVIDs (mm)	15.4	19.8	16.6	19.9	19.6
LVPWs (mm)	9	9.6	10.1	11.8	10.1
FS (%)	39.05	35.99	44.8	38.71	42.5
EF (%)	71.7	67.1	77.5	70.39	74.7
LVIDdn	1.7	1.7	1.96	2.06	1.91
E/A ratio	1.98	2.8	1.5	1.08	1.67
S′ (cm/s)	9.7	10.8	7.2	8	-
E′/A′	1.71	1.46	0.79	0.9	-
Qp:Qs	2	0.94	1.5	2	1.7

**Table 2:** Blood test of cardiac marker and complete blood count.

	Case 1	Case 2	Case 3
**Biochemical blood test**			
ProBNP (pmol/L)	425	820	-
Troponin I (ng/mL)	<0.2	<0.2	-
Creatine kinase (U/L)	200	113	-
**Complete blood count**			
Hct (%)	36.9	46.1	46
RBC (×10^6^ L/µL)	5.35	7.11	6.62
HGB (g/dL)	12.3	15.1	15.2
WBC (×10^3^ L/µL)	17.06	9.18	8.06
Platelet (×10^3^ L/µL)	392	344	291

ProBNP: Pro-Brain natriuretic peptide; Hct: Hematocrit, RBC: Red blood cell; HGB: Hemoglobin; WBC: White blood cell.

## Data Availability

Data supporting the findings of this study are available from the corresponding author upon reasonable request.

## Abbreviations

The following abbreviations are used in this manuscript:

ARVF	aorto-right ventricular fistula
LA	left Atrium
LV	left ventricle
LVIDdN	left ventricular internal dimension in diastole
PDA	patent ductus arteriosus
Qp:Qs	pulmonary-to-systemic blood flow ratio
RV	right ventricle
SOVA	sinus of Valsalva aneurysm
VSD	ventricular septal defect
